# Perceived facilitators and barriers to healthy dietary behaviour in adults with type 2 diabetes mellitus in Kenya: a qualitative study

**DOI:** 10.1017/S136898002200221X

**Published:** 2022-12

**Authors:** Moses Mokaya, Eddah Saruni, Florence Kyallo, Roman Vangoitsenhoven, Christophe Matthys

**Affiliations:** 1 Department of Human Nutrition Sciences, Jomo Kenyatta University of Agriculture and Technology, Nairobi, Kenya; 2 Experimental and Clinical Endocrinology, Department of Chronic Diseases and Metabolism, KU Leuven, Herestraat 49, 3000 Leuven, Belgium; 3 Department of Community Health Nursing, Jomo Kenyatta University of Agriculture and Technology, Nairobi, Kenya; 4 Department of Endocrinology, University Hospitals Leuven, Leuven, Belgium

**Keywords:** Dietary behaviour, Health, Low- and middle-income country, Type 2 diabetes mellitus

## Abstract

**Objective:**

This study aimed to explore the facilitators and barriers to healthy dietary behaviour in adults with type 2 diabetes mellitus (T2DM) in Kenya.

**Design:**

A qualitative descriptive design using telephone interviews was applied. An interview guide was developed through a modified theoretical framework.

**Setting:**

This study was conducted in selected hospitals in Nakuru County, located in west-central Kenya.

**Participants:**

A two-step sampling strategy was used to select hospitals and study participants. Adult participants aged 30 to 85 years, with T2DM from six hospitals were selected based on their ability to openly elaborate on the theme of dietary behaviour.

**Results:**

Thirty respondents were interviewed (mean age 62 years; 43·3 % females). The average duration of the interviews was 32:02 min (sd 17·07). The highest-ranking internal facilitators of healthy dietary behaviour were knowledge of healthy food choices, gardening, self-efficacy, food preparation skills and eating at home. External facilitators included inaccurate beliefs and information on food and diet, education by healthcare workers, food availability, proximity to food selling points and family support. Internal barriers included tastes and preferences, health conditions barring intake of certain foods, and random eating of unhealthy foods. External barriers included socio-economic factors, seasonal unavailability of fruits and food safety concerns.

**Conclusions:**

Facilitators and barriers to healthy dietary behaviour among Kenyan adults with T2DM are related to food literacy and include selection, preparation and eating. Interventions to enhance healthy dietary behaviour should target context-specific knowledge, skills and self-efficacy.

Type 2 diabetes mellitus (T2DM) is the most common type of diabetes, contributing to 90 % of all diagnosed cases globally^(1)^. Worldwide, 76·3 % of people with diabetes are living in low- and middle-income countries. In Kenya, 2·2 % of the adults have diabetes and 36·0 % of the adult population have undiagnosed diabetes^(2)^. Further, Kenya records the second highest number of deaths in Africa that are related to T2DM for people aged below 60 years. In 2019 alone, the national healthcare expenditure related to diabetes in Kenya was USD 70·5 Million^(3)^, which equates to USD 324 per person with T2DM^(2)^.

The rising prevalence of T2DM in low- and middle-income countries is associated with the nutrition transition, urbanisation, cultural and social changes, sedentary lifestyles, and changes in diagnostic criteria and screening practices^(4–6)^. This notwithstanding, healthy dietary intake is an essential self-care behaviour in the management of diabetes to optimise glycaemic control^(7)^. In Africa, numerous factors have been attributed to unhealthy dietary practices in people with T2DM. These factors include poor access to quality food, Western cultural influences, low-quality healthcare, poverty, educational level and perceptions about the disease^(8)^. To influence healthy dietary practices in people with T2DM in low- and middle-income countries, there is a need for a clear insight into the broad sociocultural aspects, including cultural beliefs, and family and communal relations of the patients^(5)^. Evidence shows that higher socio-economic status or residence in urban settings is associated with higher energy intake, cholesterol and saturated fat intake^(9)^.

It has been argued that nutrition knowledge is a necessary but not a sufficient factor for changes in consumers’ food behaviours^(10)^. However, a recent cross-sectional study in Kenya revealed that dietary knowledge, level of education, occupation and income influence dietary practices in people with T2DM^(11)^. Besides these population-based findings, international guidelines recommend individualised assessment for adults with diabetes^(12–14)^. We, therefore, sought to identify individual-level information in uncontrolled and context-specific settings, through a qualitative study.

This study aimed to explore the perceived facilitators and barriers to healthy behaviour in adults with T2DM in Kenya.

## Methods

The consolidated criteria for reporting qualitative research (COREQ) guided reporting of this study (online Supplementary File 1)^(15)^.

### Study design and participants

A qualitative descriptive study using telephone interviews was conducted between August and October 2020.

This study was conducted in Nakuru County, located in west-central Kenya. The study site was selected given that it is a cosmopolitan population that includes rural and urban settings. Nakuru County has a population of 2·16 million people with an estimated prevalence of T2DM of 2·4 % in adults aged more than 50 years^(16,17)^.

A two-step sampling strategy was used to select hospitals and study participants. In the first step, six hospitals were purposively selected to include a mix of urban and rural hospital settings, as well as public and private hospitals. In the second step, participants with the capability to openly elaborate on the subject matter of the study were selected with the support of hospital healthcare personnel. A sample size of thirty participants was determined based on guidance that qualitative studies reach saturation after twenty interviews^(18,19)^.

Participants met the inclusion criteria if they were aged 30–85 years, diagnosed with T2DM receiving diabetes care in the selected hospitals, understood and spoke English or Kiswahili and could receive calls on a mobile phone^(20)^.

This study received ethical approval from the AMREF Health (African Medical Research Foundation) Ethical and Scientific Research Committee (Approval Number ESRC P752/2020). Additionally, a research permit was received from the National Commission for Science Technology and Innovation (License No: NACOSTI/P/20/4518) and the authority to conduct research from the County Government of Nakuru. Due to the COVID-19 restrictions, verbal informed consent was electronically recorded at the start of all telephone interviews.

### Theoretical framework

**Fig. 1:**
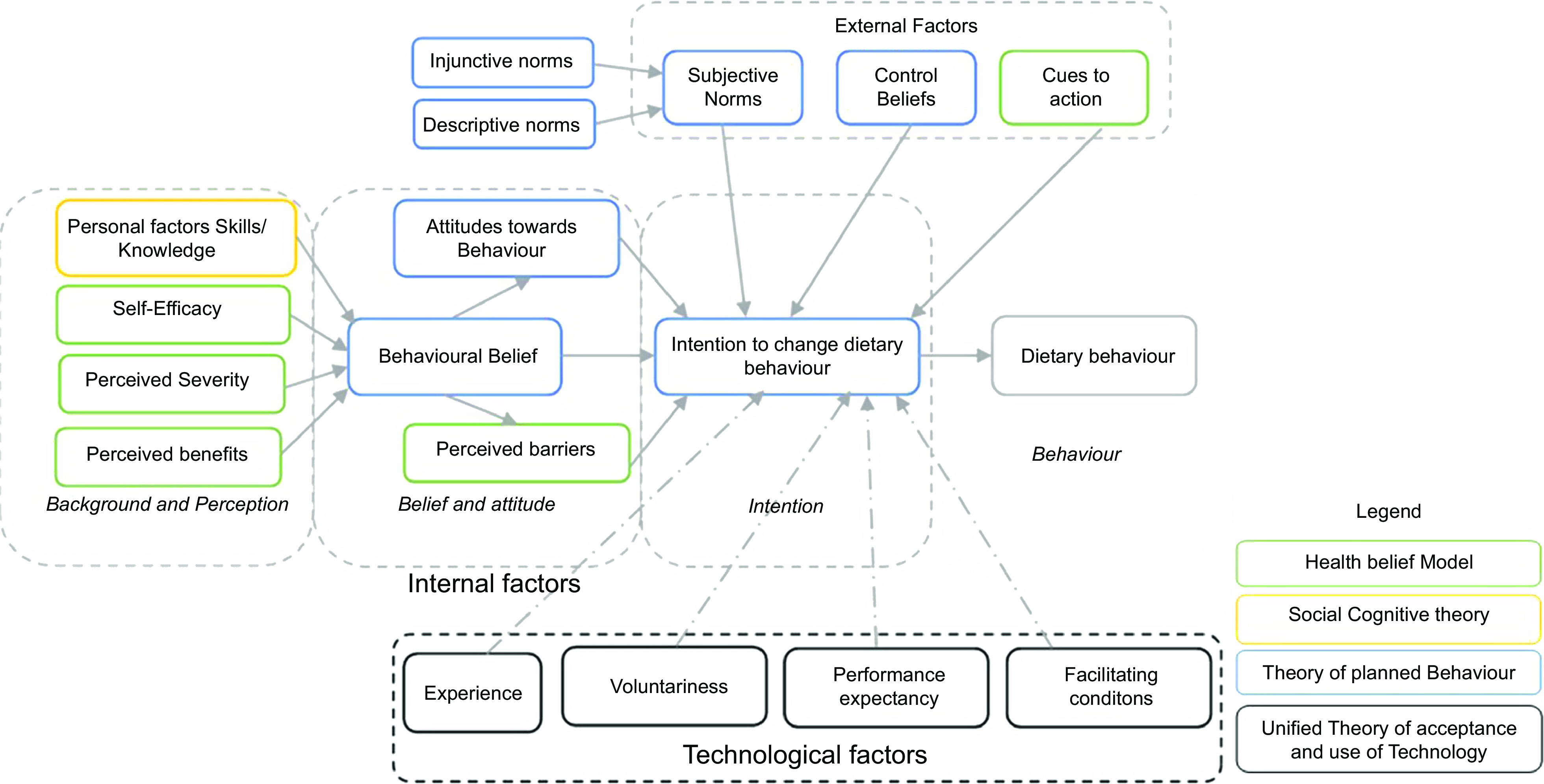
Modified theoretical framework for the development of telephone interviews

A modified theoretical framework (Fig. 1) guided the development of the telephone interview guide. In this framework, dietary behaviour is driven by a combination of internal, external and technological factors. Internal factors are defined by constructs in the health belief model, social cognitive theory and theory of planned behaviour^(21–23)^. External factors include subjective norms and control beliefs in the theory of planned behaviour and cues to action from the health belief model^(21,22)^. Technological factors are described by constructs in the unified theory of acceptance and use of technology, including experience, voluntariness, performance expectancy and facilitating conditions^(24)^.

According to this framework, the background and perceptions influence behavioural beliefs. The background and perception include knowledge and skills, self-efficacy, perceived severity of disease and perceived benefits to dietary behaviour. The behavioural belief, perceived barriers and attitudes towards dietary behaviour have an impact on the intention to change behaviour. Dietary behaviour may also be influenced by subjective norms, control beliefs and cues to action, all of that individually or cumulatively affect the intention to change behaviour. Subjective norms include injunctive and descriptive norms. Injunctive norms refer to what peers and people of importance to an individual think about the behaviour, while descriptive norms are what the significant others do that may then lead to behaviour. In contextualising the framework to present-day lifestyles, we included mobile technology as a factor that may affect dietary behaviour.

### Data collection and analysis

Telephone interviews were conducted in Kiswahili or English by two interviewers from the research team (MM and ES). MM holds an MSc in Human Nutrition and is a PhD researcher, while ES holds a BSc in Nursing and is a Registered Nurse in Kenya. MM and ES are male and female, respectively, and were both trained in Qualitative Data Collection methods.

The participants did not have any prior relationship with the interviewers before the commencement of the study. Before starting the interviews, the two interviewers were introduced to the participants through a telephone call by a community mobiliser in each of the hospitals. The community mobilisers provided the names and occupations of the interviewers and briefed respondents on the purpose of the interviews. A total of thirty participants were interviewed, and given that the current study was a one-time telephone interview, there were no participants that dropped out.

The interview guide included sociodemographic and health history data, perceived facilitators and barriers to dietary behaviour in the external and internal food environments. Before the telephone interviews, the questionnaire was piloted on three participants (10 % of the participants) drawn from one of the selected hospitals, who were not included in the actual study. The pre-test interview transcript was used to adjust the questionnaire and ensure content, semantic and conceptual equivalence^(25)^. The interviewers did not take telephone interview notes because all sessions were recorded using the Audacity® software and data were securely stored. Respondents were provided with unique identifiers for tracking the responses and ensuring anonymisation of responses. Transcription and translation of the recordings were conducted by MM and ES.

Two researchers (MM and ES) used NVivo 12 Software to code the responses from each respondent. Responses to the questions were coded through inductive thematic analysis to identify facilitators and barriers to healthy dietary behaviour. Emerging themes were then combined to develop data codes, which included quotes from the interviews, using the respondents’ unique identifiers. Similar ideas were thereafter organised into themes representing the facilitators and barriers to healthy dietary behaviour from the interviews.

## Results

A total of thirty respondents (mean age 62 years; 43·3 % females) were interviewed, with an average duration of the interview of 32:02 min (sd 17·07). The mean duration since diagnosis of diabetes of 8·50 years (sd 7·80).

### Sociodemographic and health characteristics of the participants

Table 1 summarises the sociodemographic and health characteristics of the participants. In summary, 60 % (18/30) of the respondents were aged more than 60 years old, including seventeen females (56·7 %).

**Table 1:** Sociodemographic and health characteristics of participants

Characteristic	Details	*n*	%
Age (years)	31–40	4	13·3
	41–50	1	3·3
	51–60	7	23·3
	>60	18	60·0
Sex	Male	13	43·3
	Female	17	56·7
Marital status	Never married	2	6·7
	Currently married	24	80·0
	Separated	1	3·3
	Widowed	2	6·7
Education level	No formal schooling	4	13·3
	Completed primary school	10	33·3
	Completed secondary school	9	30·0
	Completed tertiary Education	7	23·3
Employment status	Employed	5	16·7
	Self-employed	19	63·3
	Retired	5	16·7
	Unemployed	1	3·3
Average monthly income (Ksh)	Less than 5000	9	30·0
	5001–10 000	9	30·0
	10 001–40 000	9	30·0
	40 001–70 000	1	3·3
	70 001–100 000	1	3·3
	More than 100 000	1	3·3
Family history of diabetes	Yes	21	70·0
	No	9	30·0
Diagnosed with hypertension	Yes	21	70·0
	No	9	30·0

### Facilitators and barriers to healthy dietary behaviour

Table 2 describes the highly ranking themes from the interviews, based on the theoretical framework.

**Table 2:** Facilitators and barriers to healthy dietary behaviour

Factor	Category	Theme
a. Perceived facilitators	Internal factors	Knowledge of healthy food
		Gardening
		Self-efficacy
		Food preparation skills
		Eating at home or carrying home-cooked food to work
	External factors	Education by healthcare workers
		Food availability
		Proximity to food selling points
		Family support
	Technological factors	Access to information through mobile phones
b. Perceived barriers	Internal factors	Tastes and preferences
		Health conditions barring intake of certain foods
		Random eating of unhealthy foods
	External factors	Inaccurate beliefs and information on food and diets
		Socio-economic factors
		Seasonal unavailability of fruits
		Food safety concerns

### a. Perceived facilitators to healthy dietary behaviour

### Internal factors

#### Knowledge of healthy food choice

Nearly half of the respondents revealed that knowledge of healthy food was an important facilitator to healthy diets.

*‘If you eat fruits, you feel the body is going well … if we do not use fruits and greens or vegetables your blood glucose is not controlled well if you use those foods, you have good health’* (Participant 4).

#### Kitchen garden

Kitchen gardening or subsistence farming was identified as an enabler to a healthy diet.

*‘But some people consume more vegetables because they grow vegetables… vegetables are plentiful’* (Participant 23).

Gardening was also perceived to be a component of self-efficacy that facilitated healthy dietary behaviour.

*‘Some people have laziness because you can plant greens even in a sack, you put a little water and fertilizer and if you plant vegetables can help you eat healthy food and earn money. Food items on my part I have enough food and that which is on the farm’* (Participant 20)

Additionally, gardening was identified as an option to avoid unsafe or contaminated food.

*‘Farming your food is better because you will take care of it, and you will not use preservatives. But the food we buy, some of which is grown and sprayed instead of helping you is causing us problems, so if a person can farm food like vegetables, it will be safe’* (Participant 30).

However, some respondents expressed an inability to own kitchen gardens given that they resided in rental property, or in places where there was not enough space for kitchen gardens.

#### Self-efficacy

Self-efficacy was identified as an important facilitator in the determination of healthy diets, and it was driven by the desire to maintain glucose control and overall good health.

*‘I do not eat carelessly even when I go to a party I don’t eat because the food is sweet, I look at the value of that food I eat, so I will not just eat the food, I would rather eat managu (Solanum nigrum) and avoid meat’* (Participant 21).

#### Food preparation skills

The choice of healthy cooking methods, including steaming and boiling, was preferred over other less healthy methods.

*‘Sometimes I steam the vegetables, I do not want it to cook too much, so I do not boil it. Steaming does not spoil the taste of vegetables and it keeps the nutrients too’* (Participant 26)

Further, food was largely prepared by female family members.

*‘…My wife knows how to prepare healthy food…she is the one who prepares the food’* (Participant 20).

#### Eating at home or carrying home-cooked food to work

Eating at home is a behavioural choice that enabled participants to control dietary choices. Eating out or away from home was thought to compromise self-control on healthy dietary behaviour.

*‘I always eat at home, and I am satisfied…. the food we buy out is served in small portions…and I don’t enjoy it. If I am not able to eat from home, I pack and carry my home-made food’* (Participant 4).

However, for the five respondents that were employed, eating most of the meals at home or carrying food to work was not always practical. In such circumstances, they would eat available food that was not necessarily healthy.

### External factors

#### Education by healthcare workers

The role of healthcare personnel in educating participants on food and dietary choices emerged as an important facilitator to a healthy diet. It was perceived that information provided by the healthcare personnel was more reliable compared to other sources.

*‘There is a unit at the district hospital that deals with nutrition, when I have questions, I go and ask them questions about good nutrition…’* (Participant 22).

#### Food availability

The availability of a variety of food in the local markets and shopping centres was identified as a facilitator of healthy dietary choices. Nearly a third of the respondents agreed that various food items including commonly consumed cereals, fruits and vegetables are readily available in the local markets.

*‘There are other foods we don’t grow on our farm, so we buy them mostly from the local open-air market, where they are always available’* (Participant 4).

#### Proximity to groceries

Access to groceries, food markets or food outlets within walking distance was identified as a facilitator. Some participants used either *boda-bodas* (motorcycle taxis) to reach a market or groceries that were distant, in a bid to access a wider variety of food in food outlets or groceries.

*‘To go to the market where I buy food, sometimes I can use the motorcycle taxi, or go on foot, I live close to the open-air market where I buy food’* (Participant 19).

#### Family support

Support from the family and the home food environment emerged as important facilitators. The family support described by the participants includes the selection of food and sharing of family meals as opposed to preparing special meals for persons with diabetes.

*‘For my family, food is prepared based on my needs, because of diabetes, so we eat the same food. My family has no problem with this kind of food, and they are used to it…’* (Participant 28).

### Technological factors

#### Access to information through mobile phones

Mobile telephones were described as facilitators of access to information on healthy dietary choices, either through calls or text messages. However, lack of access to the internet and technical literacy reduced the use of smartphones among older adults.

*‘I get information on diabetes or contact my doctor by SMS or by a voice call*’ (Participant 13).

### b. Perceived barriers to healthy dietary behaviour

### Internal factors

#### Tastes and preferences

It was inferred that the tastes of some foods that are healthy are not appealing to some participants. Specifically, the monotony of vegetables was mentioned, which could be associated with food preparation skills.

*I don’t like the taste of some of the food…. like sometimes I don’t feel the desire to eat vegetables because the taste is not appealing… (Participant 10).*

#### Health conditions barring intake of certain foods

Some participants identified certain health conditions as a deterrence to healthy dietary behaviours. For instance, the digestion of vegetables and ulcers were mentioned by some participants.

*‘… since the seventies, I have not eaten kales because my stomach cannot digest the vegetables. When I go to the toilet, I pass whole pieces of vegetables as I chewed by my teeth’* (Participant 11)

#### Random preferences for unhealthy foods

It was revealed by some participants that at times, they would randomly eat unhealthy foods. Specifically, the awareness of circumstances that lead to unhealthy food preferences is associated with psychological stress and poor access to healthy food. However, even with the random preferences, it was apparent that the participants were aware of the healthy dietary options for better glycaemic control.

*‘You know sometimes you find yourself eating unhealthy food I see if that’s what makes it worse or if you have a lot of thoughts (stress). With a good balance of food, it goes well but sometimes you cannot explain, maybe at that time there is a lower supply of vegetables, but if there is a good supply of vegetables it just goes well….’* (Participant 29)

### External factors

#### Incorrect beliefs and information

Despite the general knowledge on dietary management of diabetes, it emerged that some participants held incorrect beliefs and information on food choices. The beliefs were associated with cultural perceptions that were either influenced by injunctive or descriptive norms as described in our theoretical framework.

On the one hand, injunctive norms were driven by what other people in the community thought about vegetables.

*‘We the (one of the communities in Kenya) only recently we knew about vegetables like kales. Previously we knew vegetables as cattle feed…vegetables like black nightshade and amaranth we knew it is vegetables for the poor people. We grew up eating only meat and not vegetables like rabbits. We did not know that vegetables contain substances that are healthy for our body*’ (Participant 23).

On the other hand, descriptive norms were noted by the reliance of some of the respondents on their spouses’ dietary choices.

*‘Usually what I prepare for my husband is what we eat, and he likes what I cook. …after all he has been eating the food, I have prepared for the very many years we have been married’* (Participant 13).

Another male respondent stated that the spouse believes the fruit is reserved for children. This opinion also highlights how gender roles influence dietary behaviour.

#### Socio-economic factors

Low-income levels emerged as a high-ranking external barrier to healthy dietary behaviour. Low income was reported by six respondents to result in less healthy dietary choices despite the availability of food in the markets.

*‘Most people understand the food that is needed to control diabetes, but the problem is money because these foods are sold, and you are probably in the countryside, and you can’t grow them in your gardens’* (Participant 18).

#### Seasonal unavailability of fruits

The seasonal unavailability of some fruits and limited variety in the local groceries made the fruits to be more expensive. This unavailability of the fruits was thought to result from most of the fruits being imported from other neighbouring counties in Kenya.

*‘I have food available, things like fruits are scarce because some fruits are seasonal, and the available ones are in the town and town market, and I may go there once a month. And even if you want to save it, it’s hard’* (Participant 15).

#### Food safety concerns

Food safety was identified as a barrier related to the preservation and contamination of food. Unscrupulous businessmen and unregulated food markets were identified as a probable and uncontrollable avenue through which unsafe food gets to food markets and outlets.

‘*The very thing that has ruined our people is the desire for money, you will find someone who harvests maize and can’t dry it well. So, they sell it with moisture, they get more wealth and money, but put people’s lives in danger’* (Participant 27).

## Discussion

The results of this qualitative study revealed interrelated perceived facilitators and barriers to healthy dietary behaviour in adults with T2DM in Kenya.

Overall, most participants demonstrated a good understanding of healthy and unhealthy dietary behaviours for optimal management of T2DM. Respondents largely received information on healthy diet and nutrition from healthcare providers in the hospitals. However, to the best of our knowledge, the knowledge of hospital personnel on dietary practices for T2DM in Kenya has not been evaluated. There is however evidence suggesting that supporting providers with culturally adopted resources on diabetes care can improve knowledge of self-care practices in patients with diabetes^(26)^. This notwithstanding, in sub-Saharan Africa, more funding is allocated to promote education on communicable diseases like HIV, TB and malaria, as compared to non-communicable diseases^(27)^.

Kitchen gardens were identified as an enabler to the participants’ control over the type and quality of food. Specifically, most participants reported that the kitchen garden contributed to the reliable availability of vegetables. Our findings partly concur with a review that linked gardening to increased fruit and vegetable consumption, better access to healthy foods and greater value in cooking^(28)^. A recent Ugandan study also demonstrated that home gardens and urban farming could improve the availability and access to healthier, and environmentally sustainable plant-based diets^(29)^. Besides the access and availability of healthy foods, kitchen gardens also provide an opportunity for increased physical activity, both of which were associated with improved glycated Hb in a community garden project^(30)^.

Food preparation skills, a component of food literacy, contributed to healthy dietary habits^(31)^. Importantly, we found that food preparation activities were largely performed by women, and as socially assigned gender norms. Despite unique variations in cultures, gender norms have been associated with health literacy and self-efficacy that result in healthy eating in Japan^(32)^. Further, our findings also showed that perceived self-efficacy is a facilitator of a healthy diet choice and food choices. Self-efficacy is a predisposing factor that can be worsened in chronic diseases like diabetes^(33)^. However, recent evidence from a Chinese study demonstrates that both higher levels of social support and diet self-efficacy are correlated with higher diet self-management^(34)^. These findings are important in our context and highlight a potential family-based approach that can be used to enhance adherence to healthy dietary behaviour. Food preparation is however affected by the selection of food, which influences the type of foods consumed^(35)^. Further, eating from home or carrying home-cooked meals was identified as a facilitator to healthy eating. However, given that most of the respondents in the present study were elderly, it would be expected that eating from home would be more likely than eating out. Eating home-cooked meals has previously been linked to better dietary quality^(36)^.

Food availability and proximity to food outlets also emerged as facilitators of food access. However, as expected, access to healthy food was linked to economic capability. Specifically, low-income levels among our participants were cited as limiting access to healthy food, even when the food was readily available in food outlets. As it has been shown in other settings, disparities in the availability of healthy food may be a barrier to diabetes self-management^(37)^. As demonstrated in the food environment framework, socio-economic status influences access to food, which in turn impacts dietary diversity^(38)^. Additionally, inequality, poverty and unemployment are associated with a low intake of fruits and vegetables among persons living in low-income settings^(39)^. Social conditions are also known to increase the risk of non-communicable diseases either through unhealthy behaviours or through the effects of psychologically stressful livelihoods^(40)^.

In the current study, inaccurate beliefs and information on various foods were identified as a barrier to a healthy diet. Specifically, cultural beliefs about vegetables and fruits influenced a healthy diet, and fruits are seen as food for children and vegetables as food for poor people. Our findings relate to evidence showing that environmental determinants of unhealthy behaviours have been associated with accessibility, availability and cultural values^(41)^. Further, income levels, prices and availability of food, consumer preferences, home production and intra-household decision-making are determinants that have been shown to influence fruit and vegetable consumption patterns^(42)^. Yiga *et al.* have also reported cultural beliefs as influencers of dietary behaviours in urban Uganda^(43)^. The complexity of food preferences and choices, which include perceptions of health and nutrition, psychological influences, sociocultural drivers, sensory preferences, and ethical concerns, have recently been described in a systematic review in low- and middle-income countries^(44)^. Interventions targeting dietary behaviour should therefore seek to bridge gaps in sociocultural misconceptions, and financial and time limitations through knowledge, skills and self-efficacy^(43)^.

### Strengths and limitations

The strength of this study is the basis of a theoretical framework and application of a structured interview guide with open-ended questions. However, we acknowledge that this study was limited by three factors. First, our study focused on perceived aspects of individuals and did not include family members given that we used telephone interviews. Including family members would provide a broader perspective on facilitators and barriers to healthy eating habits and food choices. Secondly, we used telephone interviews that may be biased through the absence of visual cues, data loss and message content distortion^(45)^. Thirdly, we conducted interviews with thirty participants, most of whom were elderly. As such, as expected in qualitative studies, our findings are not generalisable but provide a contextual understanding that is important for designing experimental studies in the same settings. Despite the attainment of saturation, this sample may not be representative of the Kenya population with T2DM.

## Conclusion

Healthy dietary behaviour is facilitated and deterred by intertwined factors related to food literacy. Specifically, selection, preparation and eating of food broadly emerged as leading themes in the current study. Context-specific interventions targeting knowledge, skills and self-efficacy provide an opportunity to enhance the facilitators and mitigate the barrier to healthy dietary behaviour.

## Supplementary material

For supplementary material accompanying this paper visit https://doi.org/10.1017/S136898002200221X. click here to view supplementary material
